# Foreign modeling agent reaction; managed with two staged surgical reconstruction. Case report and review of literature

**DOI:** 10.1016/j.ijscr.2021.106137

**Published:** 2021-06-29

**Authors:** Félix Alejandro Pérez Tristán, Félix Pérez-Rocha, Carlos Pérez Tristán, Arely Fernanda Tamariz Campillo, Raúl Alexander Cuevas Bustos, Maria Fernanda Castillo De la Rosa, Marcos Jafif Cojab

**Affiliations:** a Hospital Civil de Guadalajara Fray Antonio Alcalde, Department of Plastic and Reconstructive Surgery, Mexico; b Hospital Angeles Lomas, Department of Surgery, Mexico; c Hospital Angeles Lomas, Department of Internal Medicine, Mexico; d Universidad Anahuac Mexico Norte, Facultad de Ciencias de la Salud, Mexico; e Centro Medico Roca Medical Center, Monterrey, Mexico; f Tecnologico De Monterrey, Mexico

**Keywords:** Foreign modeling agent reaction, Biomaterials, Composite buttock augmentation

## Abstract

**Introduction:**

In Mexico, body modeling with injectable biomaterials such as liquid silicone is a common practice in non-certified clinics by non-medical personnel; These materials produces a series of complications described as Foreign modeling agent reaction (FMAR) with variable spectrum of severity.

**Case presentation:**

38-year-old female with history of biomaterial injection in a non-certified cosmetic clinic 10 years prior to evaluation. Presents with intermittent symptoms characterized by fever, erythema, induration and pain in the gluteal region. An exhaustive debridement and resection with primary closure was performed. Thereafter, reconstruction was done using a combined technique with gluteal implants and autologous fat graft, evolving without complications.

**Discussion:**

The use of biomaterials has been widely documented throughout history; liquid silicone being one of the protagonists. Used for aesthetic purposes and modeling areas such as buttocks and breasts. They have been associated with an assortment of early or late onset complications, sometimes resulting in fatal outcomes. Various treatment modalities have been described depending on the severity of presentation, from conservative to surgical management.

**Conclusion:**

There is a shortage of treatment guidelines regarding FMAR due to its wide variety of presentation, treatment must be individualized to obtain adequate results. Although conservative treatment has shown good results, the anatomical alterations usually condition dissatisfaction that should be addressed with reconstructive techniques [10].

## Highlights

• Body modeling with injectable biomaterials such as liquid silicone is a common practice in non-certified clinics by non-medical personnel.
• FMAR is characterized by local and systemic alterations, most patients have continuous manifestations since diagnosis.
• There is a shortage of treatment guidelines regarding FMAR due to its wide variety of presentation.

## 1 Introduction

Nowadays, standards of beauty have conditioned the development of a variety of minimally invasive techniques to improve body contour.

A common procedure is the injection of modeling substances; because it is a simple, minimally invasive, outpatient, inexpensive and painless method. Throughout history various substances have been used for this purpose. Polydimethylsiloxane or liquid silicone represents the majority of cases reported in literature. Nevertheless, studies carried out by the “*National Autonomous University of Mexico*” found a wide variety of biomaterials [1].

The reason behind silicone's use as a filling material relies in its antigenic and non-carcinogenic properties, as well as being permanent and inexpensive [2], [7].

The lack of standardization of these substances and the consequent use of non-purified or adulterated silicones by non-certified individuals, has led to an increase in associated complications. This is common in Mexico, where aesthetic pseudo-clinics with non-medical personnel are performing these procedures [1], [2].

Foreign modeling agent reaction (FMAR) has been defined as any clinical manifestation, local or systemic, presenting after parenteral administration of non-biodegradable substances meant to model the body [9].

The severity of complications is related to the amount and type of material used, making each case unique and treatment individual [[1], [10]].

## 2 Case report

**Fig. 1:**
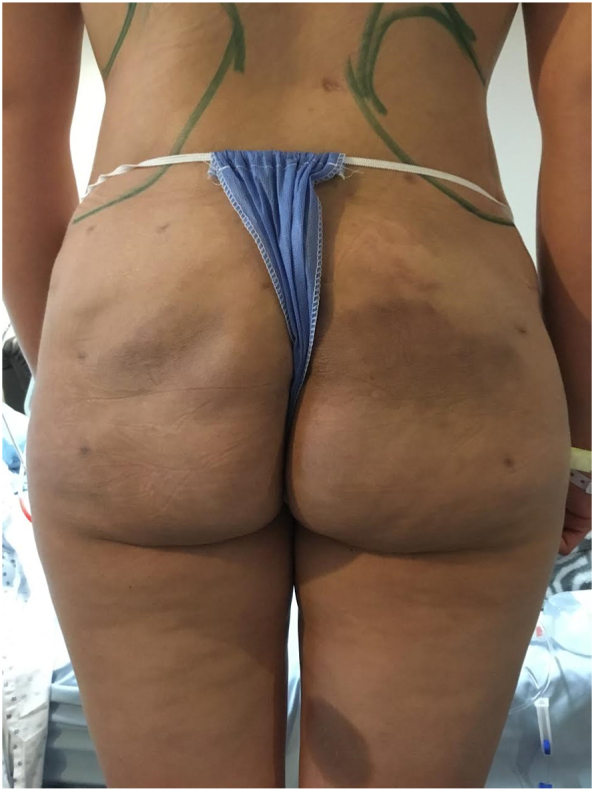
Before 1st stage surgery; Irregular induration areas, hyperpigmentation.

A 38-year-old female who came for evaluation presenting episodes of fever, chills, erythema and pain in the gluteal region, on physical examination we found irregular indurations, hyperpigmentation and asymmetry. She was intermittently treated for 5 years with nonsteroidal anti-inflammatory drugs (NSAIDs) and unspecified antibiotics. Refers having undergone into treatment injection of an unspecified biomaterial in a non-certified clinic by non-medical personnel for gluteal augmentation 10 years ago, evolving without complications for 5 years. Physical examination revealed asymmetry and multiple firm, irregular and tender subcutaneous nodules with areas of hyperpigmentation (Fig. 1).

**Fig. 2:**
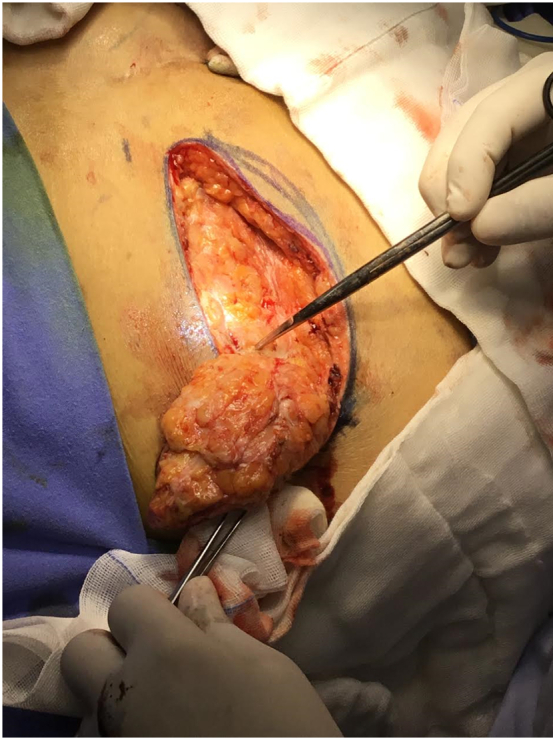
Nodular tissue and fibrosis.

**Fig. 3:**
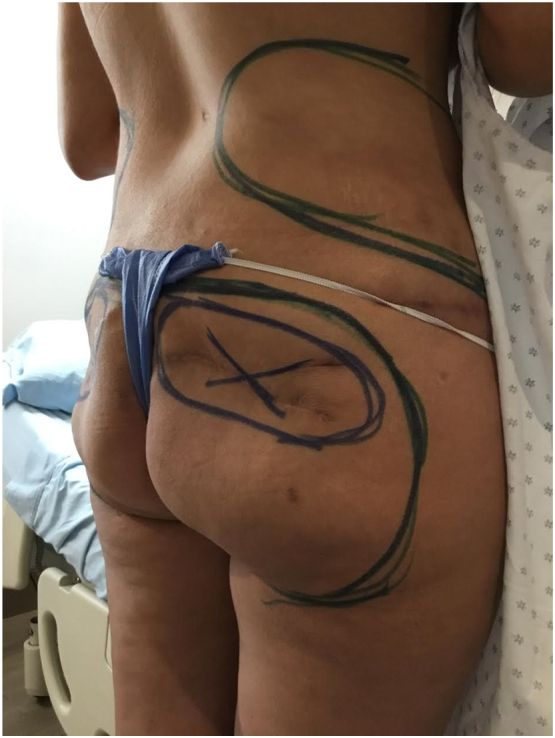
6 months after 1st stage surgery.

**Fig. 4:**
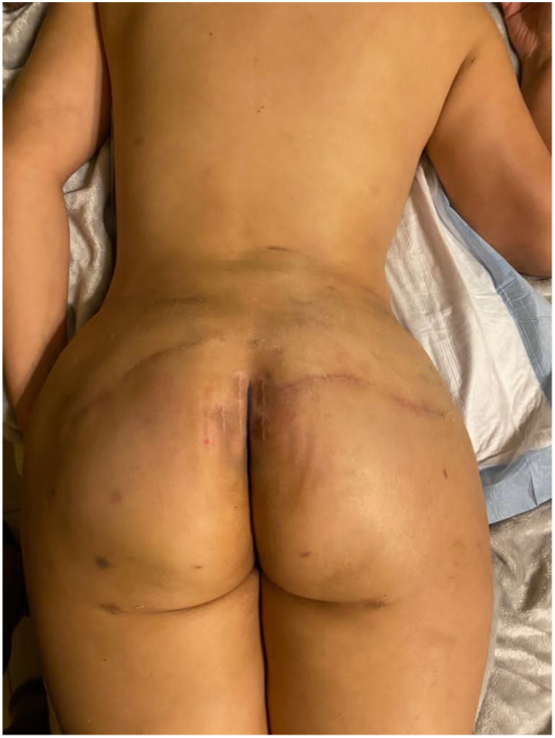
Reconstruction results with composite technique 6 months post op.

Initially the patient received conservative medical treatment with NSAID's, corticosteroids and systemic antibiotics, which had only partial improvement of symptoms but without changes in the site of injury. We used doxycycline associated with clindamycin for seven days without success. A two-stage reconstructive treatment was planned; In the first stage, an exhaustive debridement and resection of a dense and nodular fibrotic tissue along with subcutaneous fat and skin respecting muscular fascia, about 270 g were removed, leaving healthy muscular tissue (Fig. 2) and a primary closure was performed. Histopathological study was not performed. Patient was discharged only with pain medication and reschedule 6 months later for second procedure. Second stage reconstructive surgery of the residual anatomical alterations (Fig. 3) was made by means of a combined technique with intramuscular colocation of gluteal implants (300 cm^3^) and autologous fat graft (35 ml each glute) for reshaping after implant placement. Patient's evolution was followed up for 6 months without complications (Fig. 4).

## 3 Discussion

Throughout history, several products have been used to find a substance capable of providing volume and contour in areas of the body [1]. In 1948, it was proposed that silicon was physiologically inherent, and its use in pure form became popular in Germany, Switzerland and Japan [2], [3].

Due to the increasing number of complications the FDA prohibited the use of Polydimethylsiloxane as modeling material in 1991 [3]. However, some studies suggest that liquid silicon can be applied by certified injectors in selected patients with the FDA approved substances for cosmetic purposes [3]. Despite FDA's recommendations, the use of non-purified or adulterated liquid silicon is common in Mexico.

When the modeling material comes into contact with the recipient's tissue, a local inflammatory response is triggered which causes the subsequent formation of granulomas. The theory behind this formation is that an infectious process or trauma, conditions the activation of T lymphocytes and production of cytokines such as TNF alpha and pro-inflammatory interleukins. Leading to the formation of granulomas [4].

FMAR is characterized by local and systemic alterations, with nonspecific pattern of recurrence; most patients have continuous manifestations since diagnosis, but there may be cases where periods of spontaneous improvement alternate with severe exacerbations [4]. The timing of symptom onset can vary from weeks to years. Cases have been reported up to 25 years after application [4], [5].

The clinical presentation varies according to the injected material and the amount used. The most common local complications are: inflammation, subcutaneous nodules and plaques, chronic cellulitis, edema, erythema, hyperpigmentation, ulcerations, necrosis, fibrosis and fistulas [5].

Due to the migrative potential of the biomaterial, tissue changes can appear at distant sites from primary injection.

Systemic manifestations are also seen; most common are fever and malaise but granulomatous reactions like granulomatous hepatitis, renal failure, and erosive arthritis have been described [6].

Approaching these patients has become a therapeutic challenge due to the variation in degree of severity and extension thus, resulting in treatment individualization. Management with NSAIDs, corticosteroids, and antibiotics is efficient for mild cases characterized by erythema, edema, and cellulitis [6], [7]. The recommended antibiotics are tetracyclines, particularly minocycline and doxycycline. Immunomodulators such as etanercept and imiquimod cream 5% have been used with success in combination with antibiotics [6], [7].

It is important to emphasize that conservative treatment will not be able to correct resulting deformities such as subcutaneous nodularity, fibrosis and asymmetry in severe cases [7].

Surgical management is indicated for well-circumscribed lesions in which complete resection of the affected tissue is possible. Depending on the extension of resection the use of flaps or grafts for reconstruction may be necessary [8].

In cases involving extensive regions or poorly defined lesions; complete removal of the affected tissue would result in large wounds susceptible to infection and poor aesthetic outcomes. Therefore, some suggest multiple stage resections with the use of negative pressure therapy and subsequent closure and reconstruction [8].

The resultant anatomical alterations associated with either conservative or surgical resection often generate patient dissatisfaction. This is why a reconstructive procedure should be planned in order to achieve best results.

In this patient a composite buttock augmentation was performed for reconstructive purpose 6 months after primary resection; using intramuscular implant placement technique for core projection combined with subcutaneous fat grafting for remaining irregularities [9].

## 4 Conclusion

There is a shortage of treatment guidelines regarding FMAR due to its wide variety of presentation. It should be suspected in all patients with history of injection of biomaterials for cosmetic purposes in non-certified clinics. Patients must be individualized to choose best course of treatment. Conservative therapeutics have shown to be effective for cases of mild presentation. Goals of treatment are achieved by resecting all affected tissue including; nodules, inflammatory, necrotic and fibrotic tissue. We empathize that a caution resection must be made in order to avoid complications since it is well known that remaining affected tissue could reactivate the disease in the future. In this case after a week of conservative treatment and primary resection of all the affected tissue; a surgical reconstructive approach was made 6 months after first procedure using current augmentation tendencies such as the composite technique with scar correction resulting in surgeon and patient satisfaction. Patient was followed up for 6 months evolving without complications [10].

## Ethical approval

There was no need for ethical approval.

## Funding

There was no sponsorship for this study.

## Author contribution

Félix Alejandro Pérez Tristán MD Data collection, data analysis, writing the paper.

Félix Pérez-Rocha MD Data collection, data analysis, writing the paper.

Carlos Pérez Tristán MD Data collection, data analysis.

Arely Fernanda Tamariz Campillo MD Data collection, data analysis.

Raúl Alexander Cuevas Bustos MD Data collection, data analysis.

Maria Fernanda Castillo De la Rosa Data collection, data analysis.

Marcos Jafif Cojab MD Data collection, data analysis, writing the paper.

## Guarantor

Félix Alejandro Pérez Tristán MD.

## Registration of research studies

N/A.

## Consent

Written informed consent was obtained from the patient for publication of this case report and accompanying images. A copy of the written consent is available for review by the Editor-in-Chief of this journal on request.

## Declaration of competing interest

There was no conflict of interest.
